# A deep learning model enables accurate prediction and quantification of pulmonary edema from chest X-rays

**DOI:** 10.1186/s13054-023-04426-5

**Published:** 2023-05-26

**Authors:** Dominik Schulz, Sebastian Rasch, Markus Heilmaier, Rami Abbassi, Alexander Poszler, Jörg Ulrich, Manuel Steinhardt, Georgios A. Kaissis, Roland M. Schmid, Rickmer Braren, Tobias Lahmer

**Affiliations:** 1 Klinik und Poliklinik für Innere Medizin II, Klinikum rechts der Isar, Munich, Germany; 2 III. Medizinische Klinik, Universitätsklinikum Augsburg, Augsburg, Germany; 3 Innere Medizin - Gastroenterologie, Krankenhaus Agatharied, Hausham, Germany; 4 Institute for Diagnostic and Interventional Radiology, Klinikum rechts der Isar, School of Medicine, Technical University of Munich, Munich, Germany

**Keywords:** Pulmonary edema, Transpulmonary thermodilution, TPTD, Extravascular lung water, EVLWI, Chest X-ray

## Abstract

**Background:**

A quantitative assessment of pulmonary edema is important because the clinical severity can range from mild impairment to life threatening. A quantitative surrogate measure, although invasive, for pulmonary edema is the extravascular lung water index (EVLWI) extracted from the transpulmonary thermodilution (TPTD). Severity of edema from chest X-rays, to date is based on the subjective classification of radiologists. In this work, we use machine learning to quantitatively predict the severity of pulmonary edema from chest radiography.

**Methods:**

We retrospectively included 471 X-rays from 431 patients who underwent chest radiography and TPTD measurement within 24 h at our intensive care unit. The EVLWI extracted from the TPTD was used as a quantitative measure for pulmonary edema. We used a deep learning approach and binned the data into two, three, four and five classes increasing the resolution of the EVLWI prediction from the X-rays.

**Results:**

The accuracy, area under the receiver operating characteristic curve (AUROC) and Mathews correlation coefficient (MCC) in the binary classification models (EVLWI < 15, ≥ 15) were 0.93 (accuracy), 0.98 (AUROC) and 0.86(MCC). In the three multiclass models, the accuracy ranged between 0.90 and 0.95, the AUROC between 0.97 and 0.99 and the MCC between 0.86 and 0.92.

**Conclusion:**

Deep learning can quantify pulmonary edema as measured by EVLWI with high accuracy.

## Introduction

Pulmonary edema is one of the most common findings in chest radiographs [1] and has important clinical consequences. By impeding the gas exchange and reducing lung compliance, severe pulmonary edema is potentially life threatening [2]. Measuring and monitoring pulmonary edema is useful in many, but especially important in critically ill patients.

Technically, the attenuation of X-rays should be proportional to the amount of lung water, and thus, a chest radiograph should be a valuable tool in monitoring the amount of pulmonary edema. Commonly, radiologists rate the severity on a categorical scale. A quantitative measure for pulmonary edema widely used for critically ill patients is the extravascular lung water (EVLW) which is defined as the amount of water accumulating in the lungs outside of the pulmonary vasculature [2]. Measurement of EVLW by transpulmonary thermodilution (TPTD), although invasive, shows good correlation with the gold standard ex vivo method of gravimetry [3]. However, mixed results have been reported in the literature with the grade of correlation of clinicians’ chest X-ray reports or clinicians’ scores to extravascular lung water (EVLW), ranging from good [4, 5] over modest [6, 7] to poor [8, 9].

Recently Horng et al. used a radiologist-based categorical four grade severity score to train a deep learning classification system on chest radiographs and report a high performance [10]. To our knowledge, our present study is the first to explore the usefulness of deep learning in predicting the quantitative pulmonary edema measure EVLW from chest radiographs.

## Acquisition of the chest radiographs and classification

A total of 471 images from 431 patients were acquired between 06/2014 and 09/2022 on two Carestream Health DRX-Revolution X-ray machines (120 kV, 0.6 mAs). The images were extracted in the jpg format. We used the 374 images acquired between 06/2014 and 12/2020 as the training set and 97 images from 01/2021 to 09/2022 for the test set with no patient overlap. We included patients who underwent chest radiography and a TPTD measurement within a maximum of 24 h. TPTD measurement was performed as previously reported [11, 12] and the extravascular lung water was indexed as previously reported (EVLWI, [6]).

## Deep learning model

We developed a convolutional neural network for the image classification task. For preprocessing, all images were resized to 300 × 300 pixels and the pixel values were normalized. Data were augmented using cutmix [13]. A transfer learning approach with an EfficientNet B5 backbone [14] with pretrained weights on ImageNet was used. Fine-tuning of the last feature layer was implemented in FastAI [15] using the Adam optimizer and the cross-entropy loss function. Training and testing were performed on a Nvidia Tesla K80 or T4. A result between 0 to 0.5 and 0.5 to 1 was used for the binary classification of each image. We report the accuracy, micro-averaged area under the receiver operator curve in “one vs rest” (AUROC) with confidence Interval (CI) and the Mathews correlation coefficient (MCC) as outcome measures [16].

## Patient characteristics

The patients mean age was 64.1 years, ranging from 23 to 92 years. There were slightly less females than males (37.4%). The patients stayed from 1 to 103 days on the intensive care unit (ICU), and the average time on ICU was 21.3 days. The mean EVLWI was 14.9 ranging from 5 to 42.

## Results

**Fig. 1:**
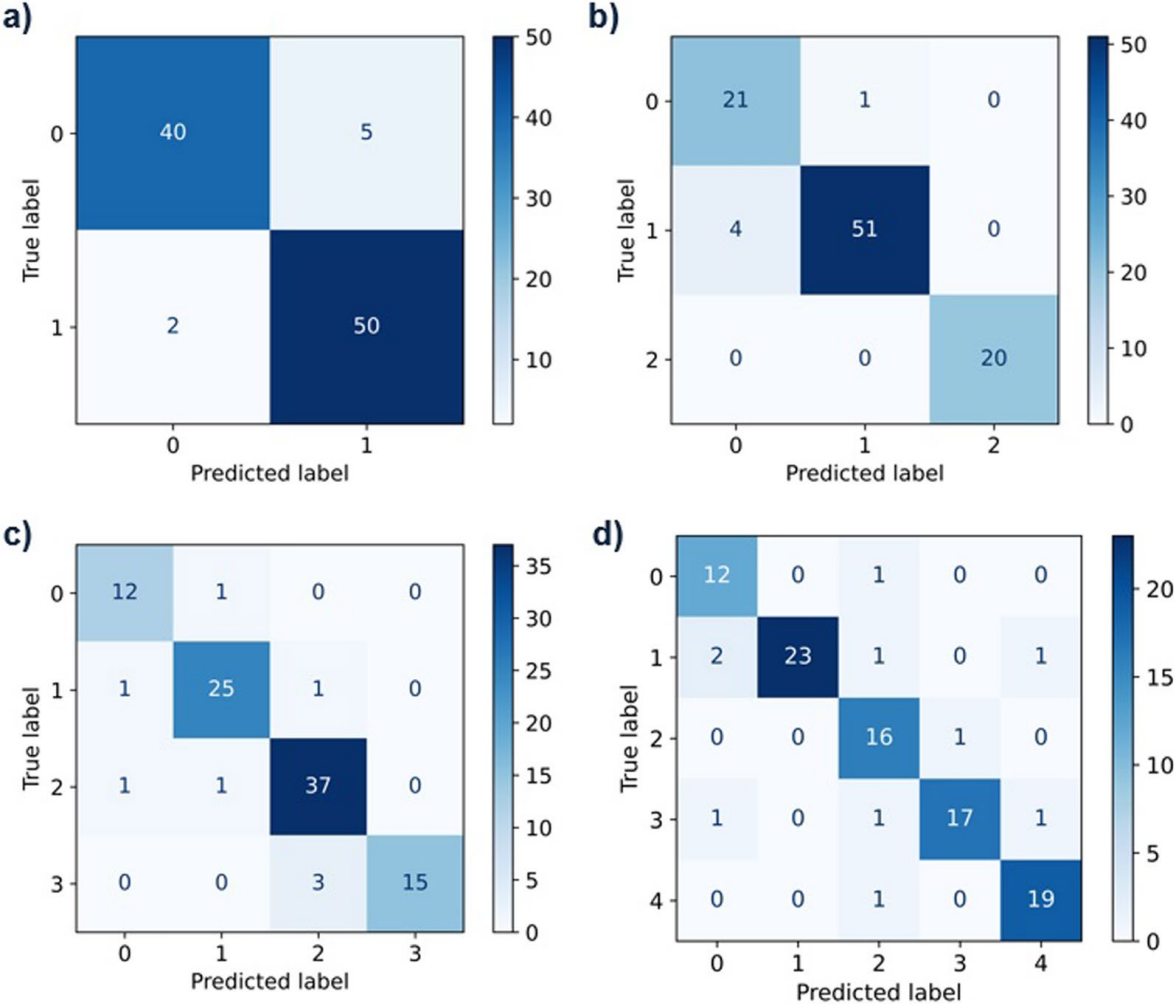
Precise prediction in the test set split into two, three, four or five classes. **a** EVLWI 0: < 15, 1: ≥ 15; ACC 0.93, AUROC 0.98, MCC 0.86; **b** EVLWI 0:[5, 12[, 1:[12, 20[, 2:[20, 44]; ACC 0.95, MCC 0.92, **c** EVLWI 0:[5, 8[, 1:[9, 13[, 2:[13, 22[, 3:[22, 44]; ACC 0.90, MCC 0.86; **d** EVLWI 0:[5, 8[, 1:[8, 12[, 2:[12, 16[, 3:[16, 20[,4:[20,44]; ACC 0.90; MCC 0.87

For the split of the test set with an EVLWI smaller than 15 and larger or equal to 15 the model reached an accuracy of 0.93, the AUROC was 0.98 (CI: [0.98, 1.00]) and an MCC of 0.86 (Fig. 1a). For the three class model we split the data into bins with an EVLWI from 5 to 11 (interval notation: [5, 12[), from 12 to 19 ([12, 20[) and from 20 to 42 ([20, 42]). The corresponding accuracy on the test set was 0.95 (Fig. 1b), the AUROC 0.99 (CI: [0.92;0.99]) and MCC was 0.92. For the four-class model we choose to split the data randomly into the following bins: [5, 8[, [9, 13[, [13, 22[, [22, 44]. The trained model reached an accuracy of ACC 0.90 (CI: [0.89; 0.97]), an AUROC of 0.99 and an MCC of 0.86 (Fig. 1c). We next split the data into five classes in the following manner: EVLWI [5, 8[, [8, 12[, [12, 16[, [16, 20[, [20,44]. The accuracy by the model was 0.90, the AUROC 0.97 (CI: [0.94; 0.98]) and the MCC was 0.87 (Fig. 1d). Splitting into six or more classes resulted in comparably diminished performance (data not shown), most likely due to the lack of training data (On average 63 images in 6 bins).

## Discussion

In this study, we sequentially developed a deep learning model that accurately quantifies pulmonary edema from chest X-ray images. We use the EVLWI measured invasively by TPTD [2] as ground truth. Our models show very good to excellent performances when binning the available data up to five classes for the clinically most relevant EVLWI range from 6 to 20.

Deep learning has been used in the literature to classify various pathologies from chest radiographs. For example, Majkowska et al. use a machine learning approach to automatically detect four abnormal findings in X-ray images [17]. For the detection of airspace opacity, which includes pulmonary edema, an AUROC of 0.91 to 0.94 is reported. Jarrel et al. use a deep learning approach to diagnose the presence of absence of congestive heart failure (CHF) from chest X-rays. The authors use a cutoff of 100 ng/L BNP as a marker for CHF and find an AUROC of 0.82 [18]. Horng et al. not only diagnose the presence but also quantify lung edema with deep learning [10]. However, the authors use radiology reports as ground truth to categorize training/test data into 4 classes ranging from “0: no edema” to “3: alveolar edema” and an AUC of 0.88 in 2vs0 and only 0.69 in 2vs1.

We see our study as an expansion of these previous works. In our opinion, there are several strong points in our approach. Pulmonary edema presents as a continuous value. We could further increase the resolution of the classification in a clinically relevant range in comparison to Horng et al.’s.

More importantly, our study uses invasively measured EVLWI values as the ground truth instead of subjectively classified radiological estimations of pulmonary edema. While there generally is a good correlation between the gold standard of gravimetry and EVLWI [3] for measuring extravascular lung water, there are mixed results in the literature for correlating classical qualitative or semi-quantitative radiological scores and EVLWI. Chrysopoulo et al. find a good correlation between a 5 scale severity score and EVLW [19]. Brown et al. report a modest positive correlation of clinician-based chest X-ray severity score and EVLW [9]. Halperin et al. describe a modest to poor correlation between a clinical edema score and an EVLW measurement [7].

There are also strong points from a conceptual view. While measuring lung water by TPTD needs a dedicated catheter and equipment, our method uses chest X-rays, which is a widely available tool. On the one hand, this could allow using EVLWI guided fluid therapy on intensive care units where TPTD is not available. On the other hand, this approach could enable access to EVLWI surrogate measurement for a much larger patient cohort. One could speculate for example guiding the diuretics dose by EVLWI in patients with heart failure.

There are limitations to our study too. While Jarrel et al. use 103,489 and Horng et al. 369,071 X-rays to test and train their models we could only use 471 images. This is due to the fact, that thermodilution is an invasive modality, feasible almost only in intensive care units. Furthermore, we tested our model only on a single institution’s critically ill patients. Thus, our results need external confirmation, despite promising results of the above-mentioned studies and the prediction of semi-quantitative scores. Finally, only imaging data acquired on our in-house portable X-ray systems was used in this study. Therefore, model generalization may require not only external imaging data but also additional training with imaging data acquired on standard up right X-ray systems.

Despite these limitations our study demonstrates, that deep learning is a useful tool for the quantification of pulmonary edema with a meaningful resolution with high accuracy.

## Data Availability

Dataset is private and is available upon request.
